# Supplementary material for: Causal Relationship between Aging and Anorexia Nervosa: A White-Matter-Microstructure-Mediated Mendelian Randomization Analysis
Source: Biomedicines. 2024 Aug 16;12(8):1874. doi: 10.3390/biomedicines12081874 (PMC11351342; doi:10.3390/biomedicines12081874)
Supplement: Supplementary file 1 [file biomedicines-12-01874-s001.zip › Supplementary Figure/Figure S4/Singe SNP plot of MR analysis for TL to ubm-b-1772.pdf]

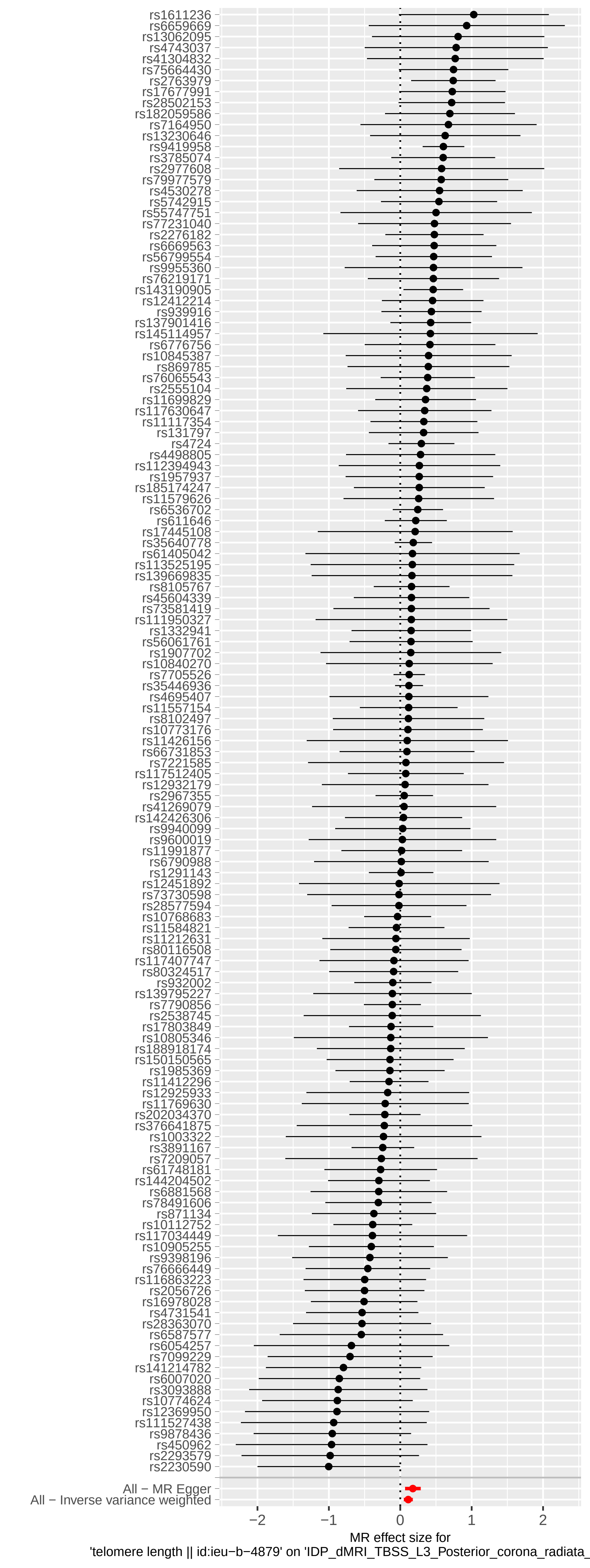

rs1611236
rs6659669
rs13062095
rs4743037
rs41304832
rs75664430
rs2763979
rs17677991
rs28502153
rs182059586
rs7164950
rs13230646
rs9419958
rs3785074
rs2977608
rs79977579
rs4530278
rs5742915
rs55747751
rs77231040
rs2276182
rs6669563
rs56799554
rs9955360
rs76219171
rs143190905
rs12412214
rs939916
rs137901416
rs145114957
rs6776756
rs10845387
rs869785
rs76065543
rs2555104
rs11699829
rs117630647
rs11117354
rs131797
rs4724
rs4498805
rs112394943
rs1957937
rs185174247
rs11579626
rs6536702
rs611646
rs17445108
rs35640778
rs61405042
rs113525195
rs139669835
rs8105767
rs45604339
rs73581419
rs111950327
rs1332941
rs56061761
rs1907702
rs10840270
rs7705526
rs35446936
rs4695407
rs11557154
rs8102497
rs10773176
rs11426156
rs66731853
rs7221585
rs117512405
rs12932179
rs2967355
rs41269079
rs142426306
rs9940099
rs9600019
rs11991877
rs6790988
rs1291143
rs12451892
rs73730598
rs28577594
rs10768683
rs11584821
rs11212631
rs80116508
rs117407747
rs80324517
rs932002
rs139795227
rs7790856
rs2538745
rs17803849
rs10805346
rs188918174
rs150150565
rs1985369
rs11412296
rs12925933
rs11769630
rs202034370
rs376641875
rs1003322
rs3891167
rs7209057
rs61748181
rs144204502
rs6881568
rs78491606
rs871134
rs10112752
rs117034449
rs10905255
rs9398196
rs76666449
rs116863223
rs2056726
rs16978028
rs4731541
rs28363070
rs6587577
rs6054257
rs7099229
rs141214782
rs6007020
rs3093888
rs10774624
rs12369950
rs111527438
rs9878436
rs450962
rs2293579
rs2230590
All − MR Egger
All − Inverse variance weighted
−2
−1
0
1
2
MR effect size for
'telomere length || id:ieu−b−4879' on 'IDP_dMRI_TBSS_L3_Posterior_corona_radiata_
